# Radiographic vertical tracheal diameter assessment at different levels along the trachea as an alternative method for the evaluation of the tracheal diameter in non-brachycephalic small breed dogs

**DOI:** 10.1186/s12917-022-03160-4

**Published:** 2022-02-01

**Authors:** Ayman A. Mostafa, Clifford R. Berry

**Affiliations:** 1 Department of Small Animal Surgery, Faculty of Veterinary Medicine, Cairo University, Giza, 12211 Egypt; 2 Diagnostic Imaging, Department of MBS, College of Veterinary Medicine, North Carolina State University, Raleigh, NC 27606 USA

**Keywords:** Radiography, VTD, Normal, Non-brachycephalic, Small breed dogs

## Abstract

**Background:**

Tracheal narrowing due to congenital tracheal hypoplasia, acquired tracheal stenosis and tracheal collapse can lead to life-threatening respiratory distress. Tracheal hypoplasia has been identified in brachycephalic dog breeds, predominantly English Bulldogs, by measuring the tracheal diameter compared to the diameter of the thoracic inlet and creating a ratio. However, reference ranges for tracheal diameter have not been established for non-brachycephalic small breed dogs. It would be advantageous to have established tracheal diameters for non-brachycephalic small breed dogs, as these are the dogs most at risk of tracheal collapse. The main objective, of this study was to radiographically evaluate vertical tracheal diameter (VTD) at three standardized locations along the trachea of non-brachycephalic small breed dogs, in an attempt to further establish a screening diagnostic protocol for canine tracheal hypoplasia. Medical records and thoracic radiographs of non-brachycephalic small breed dogs without respiratory disease were reviewed. Right lateral radiographs were reviewed. The absolute and average VTDs at three locations (location A: caudal cervical VTD; location B thoracic inlet VTD; location C: intrathoracic VTD) were standardized by manubrium length (ML), as well as by the previously utilized thoracic inlet distance (Ti-D) and proximal 3rd rib width (PR3-W) to calculate manubrium-tracheal index (M-TI), thoracic inlet-tracheal index (Ti-TI), and proximal R3-tracheal score (PR3-TS), respectively. Correlations between averaged tracheal diameter and each of the ML, Ti-D, and PR3-W, and between M-TI and each of Ti-TI and PR3-TS were calculated.

**Results:**

Eighty-one healthy dogs met the criteria for inclusion. Significant differences (*P* < 0.0001) were identified among the mean values of the absolute and standardized VTDs at levels A, B, and C. The smallest tracheal diameter was identified at the level of the thoracic inlet (Level B).

The average VTD correlated better with ML (*r*_*s*_ = 0.82, *P* < 0.0001) compared to Ti-D and PR3-W. A relatively strong correlation (*r*_*s*_ = 0.77, *P* < 0.0001) was identified between the averaged manubrium tracheal index (M-TI) and thoracic inlet tracheal index (Ti-TI).

**Conclusion:**

M-TI is an appropriate alternative to Ti-TI and PR3-TS to radiographically evaluate VTD in dogs. M-TI < 0.43, < 0.34, or < 0.38 at level A, B, or C, respectively, may indicate tracheal hypoplasia in non-brachycephalic small breed dogs. Screening of canine VTD could be achieved using M-TI.

## Background

A hypoplastic trachea is a component of brachycephalic airway syndrome affecting most likely English bulldogs and Bullmastiffs [1]; however, non-brachycephalic dogs can also be affected [2, 3]. Congenital tracheal hypoplasia and acquired tracheal stenosis, which may develop secondary to most tracheal diseases, negatively influence the general health condition and the quality of life of dogs. Furthermore, respiratory distress due to tracheal hypoplasia/stenosis or collapse is a life-threatening condition that requires proper evaluation and immediate intervention [4]. Therefore, several radiographic and CT techniques have been described to evaluate tracheal size and monitor tracheal hypoplasia in dogs [2, 5–10]. Additionally, fluoroscopy and bronchoscopy were established to diagnose and monitor dynamic tracheal collapse in dogs [8, 11]. Among these diagnostic procedures, radiography has been found to be the most widely used modality among practitioners [12, 13]. Thus, radiographic assessment of vertical tracheal diameter (VTD) was proven to be essential for diagnosing tracheal hypoplasia or stenosis and consequently for the necessity for surgery, as well as for selecting the proper sizes of the tracheal stent and endotracheal tube [8, 12, 13]. The commonly utilized radiographic procedures included measurement of the VTD at the thoracic inlet to be standardized by the thoracic inlet distance (Ti-D) [2, 6, 7, 14, 15] or the proximal 3rd rib width (PR3-W) [7, 15, 16].

To the authors’ knowledge, no previous investigation has been accomplished to radiographically compare VTD at different levels along the canine trachea using manubrium length (ML) as a normalizing parameter. Therefore, our current and upcoming studies will be comparing VTDs at three different tracheal levels using different normalizing parameters in brachycephalic and non-brachycephalic small- and large breed dogs. The objectives of the current study were to: (1) calculate manubrium tracheal-index (M-TI) for non-brachycephalic small breed dogs; (2) determine whether there is a significant difference in the standardized VTD among the three tracheal levels; (3) evaluate correlations between M-TI and each of the previously established thoracic inlet tracheal-index (Ti-TI) and proximal 3rd rib tracheal-score (PR3-TS). We hypothesize that VTD would vary according to the location of the investigated tracheal region. Our 2nd hypothesis is that M-TI could be an appropriate alternative to Ti-TI and PR3-TS in the radiographic assessment of canine tracheal index. The long-term goal is to further establish a screening diagnostic protocol for congenital tracheal hypoplasia and acquired tracheal narrowing in dogs.

## Methods

### Population

The study design was a retrospective observational investigation. Medical records and thoracic radiographs (right lateral, left lateral, and ventrodorsal views) of non-brachycephalic, small breed dogs admitted to the Small Animal Hospital at the University of Florida, College of Veterinary Medicine from November 2012 to July 2020 were retrieved. The selected population included client-owned dogs with no history or concurrent clinical or radiographic signs of cardiovascular or respiratory diseases. Any patients with heart murmur or gallop rhythm on auscultation were excluded. Radiographically, there were no detectable abnormalities associated with the respiratory tract or the cardiovascular and pulmonary structures. Subjects with geriatric pulmonary, airway, and/or vascular changes (i.e. fibrosis or mineralization/osteomas), as well as with a history of oral, neck, or chest surgery were excluded from the investigation. The investigated thoracic radiographic views were assumed to be taken at the time of peak inspiration and without sedation or anesthesia. Dogs with clinical and/or radiographic evidence of tracheal hypoplasia, thickened soft palate, redundant dorsal tracheal membrane, or esophageal abnormalities (i.e. esophageal neoplasia or foreign body, hiatal hernia, megaesophagus, or even fluid-filled esophagus) were excluded from the study. Further, dogs with thoracic vertebral anomalies or abnormally shaped, fused, or short manubrium [17] were excluded. The manubrium was considered short if its length was equal to or shorter than the corresponding second sternal segment. Right lateral thoracic radiographs on which the caudal cervical tracheal region (including C5) or the cranial portion of the manubrium could not be identified were also excluded.

### Radiographic measurements

The investigated thoracic radiographs were standardized using standard exposure factors and a focal spot-film distance (FFD) and approved in terms of quality and positioning by a board-certified radiologist (CRB). All radiographs were obtained with a digital radiography plate (CXDI-50G digital plate, Canon USA Inc., Lake Success, NY) and retrieved with an image archiving communication system (Merge PACs, Merge Healthcare Inc., Chicago, Ill) and a medical workstation. All radiographic measurements were performed on the right lateral thoracic radiographic view by the same investigator (AAM) using the same image archiving PACs system.

**Fig. 1:**
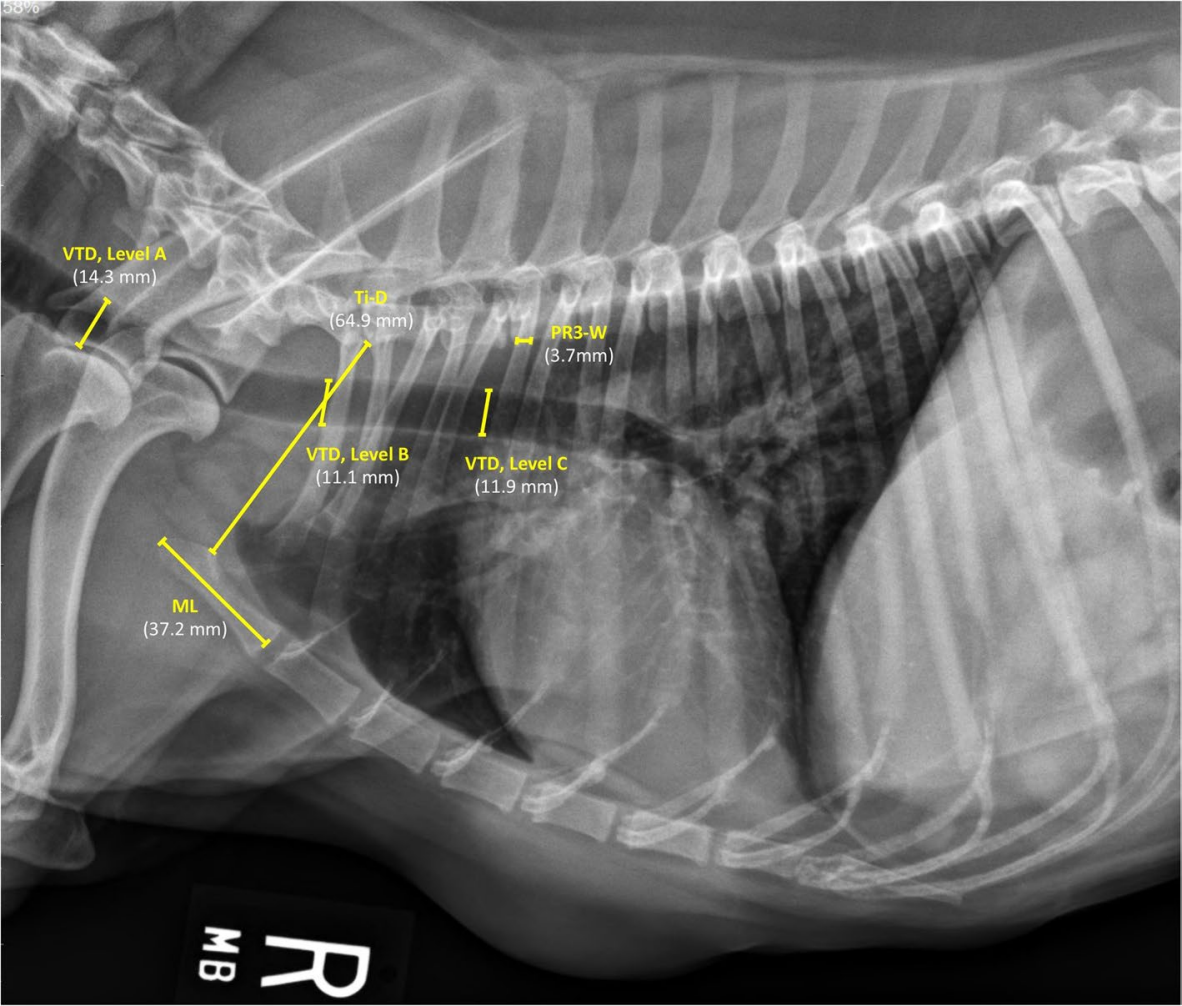
Right lateral thoracic radiographic view of a clinically normal Miniature Pinscher dog illustrating measurements of vertical tracheal diameters (VTDs) at caudal cervical (level **A**), thoracic inlet (level **B**), and intrathoracic (level **C**) tracheal regions, and measurements of manubrium length (ML), thoracic inlet distance (Ti-D), and proximal 3rd rib-width (PR3-W) for determination of manubrium (M-TI) and thoracic inlet-tracheal indices (Ti-TI) and proximal R3-tracheal scores (PR3-TS) for each absolute and average tracheal diameters

Vertical tracheal diameter (VTD) was assessed at three different levels (A, B, and C) along the trachea on the right lateral thoracic radiographic view (Fig. 1). The caudal cervical VTD was measured at the level of the mid-5th cervical vertebra (level A). The thoracic inlet VTD was measured at the level of the caudal portion of the 7th cervical vertebra (level B). The intrathoracic VTD was measured at the mid-point between the thoracic inlet and carina, at the level of mid T2 to mid T3 (level C). The three tracheal regions utilized to assess each corresponding VTD were selected and modified from previously established procedures [5, 7–9, 16, 18]. To mitigate the differences in the VTD related to interbreed variation, each absolute and average value of VTD was standardized by the corresponding manubrium length (ML), thoracic inlet distance (Ti-D), and proximal 3rd rib width (PR3-W) (Fig. 1). Elongated, bullet-shaped, rectangular, and camel head–neck–shaped manubriums were considered acceptable for ML measurement [17, 19]. The measured thoracic inlet distance (Ti-D) was modified from previous reports [2, 6, 7, 14, 20], and defined in the present study as the distance extending from the cranio-ventral aspect of the 1st thoracic vertebra to the craniodorsal manubrium at its highest point, the point of the minimum distance of the thoracic inlet. The width of the proximal third of the 3rd rib (PR3-W) was measured along the ventral aspect of the 3rd thoracic vertebra [7, 16]. Manubrium-tracheal index (M-TI = VTD: ML), thoracic inlet-tracheal index (Ti-TI = VTD: Ti-D), and proximal R3-tracheal score (PR3-TS = VTD: PR3-W) were then calculated at each tracheal level.

### Statistical analysis

Variables were assumed to be normally distributed based on the central limit theorem [21], and consequently, data analyses were performed using parametric statistical tests. Data were reported as means ±SDs and a 95% confidence interval (CI) was calculated for each variable. Variables of interest were compared using ANOVA, as well as with unpaired, 2-tailed t-test, and statistical significance was set at a *P-*value < 0.05. The Spearman’s rank correlation coefficient was calculated to evaluate the association between the average tracheal diameter and each of the manubrium length (ML), thoracic inlet distance (Ti-D), and proximal R3 width (PR3-W). Also, the correlations between the manubrium tracheal index (M-TI) and each of the thoracic inlet tracheal index (Ti-TI) and proximal R3 tracheal score (PR3-TS) were calculated using the same statistical test. All data analyses were carried out using commercially available statistical software (GraphPad Prism version 8.0.0 for Windows, San Diego, California, USA).

## Results

### Population

Medical records and thoracic radiographs of 87 non-brachycephalic, small breed dogs were reviewed. Five out of the 87 dogs (5.7%, one each of Jack Russell, Schnauzer, and Shiba Inu, and two Chinese Crested) had short manubrium and a Poodle (1.1%) had fused manubrium. The six dogs were therefore excluded from the investigation. Thus, a total of 81 dogs met the criteria for inclusion in the present study. Investigated dogs were admitted to our clinic for reasons other than respiratory or cardiovascular diseases, most likely for routine assessment of pulmonary metastasis, and for which clinical and radiographic examinations of all subjects revealed no abnormalities. The mean (±SD) values of the age and body weight were 9.4 (±3.4) years and 10.2 (±5.5) kg, respectively. Dog breeds included: 19 (23.5%) Poodles; 10 (12.3%) each of Jack Russell Terriers and Miniature Schnauzers; 9 (11.1%) Dachshunds; 7 (8.6%) Beagles; 6 (7.5%) Italian Greyhounds; 4 (5%) each of Cocker Spaniel, Pembroke Welsh Corgi, and Miniature Pinschers; 3 (3.7%) each of Shiba Inu and Chinese Crested; and 2 (2.5%) Scottish Terriers. Among the 81 dogs, there were 43 males (37 castrated) and 38 females (35 spayed), with a male to female ratio being 1.2:1.

### Radiographic measurements

**Fig. 2:**
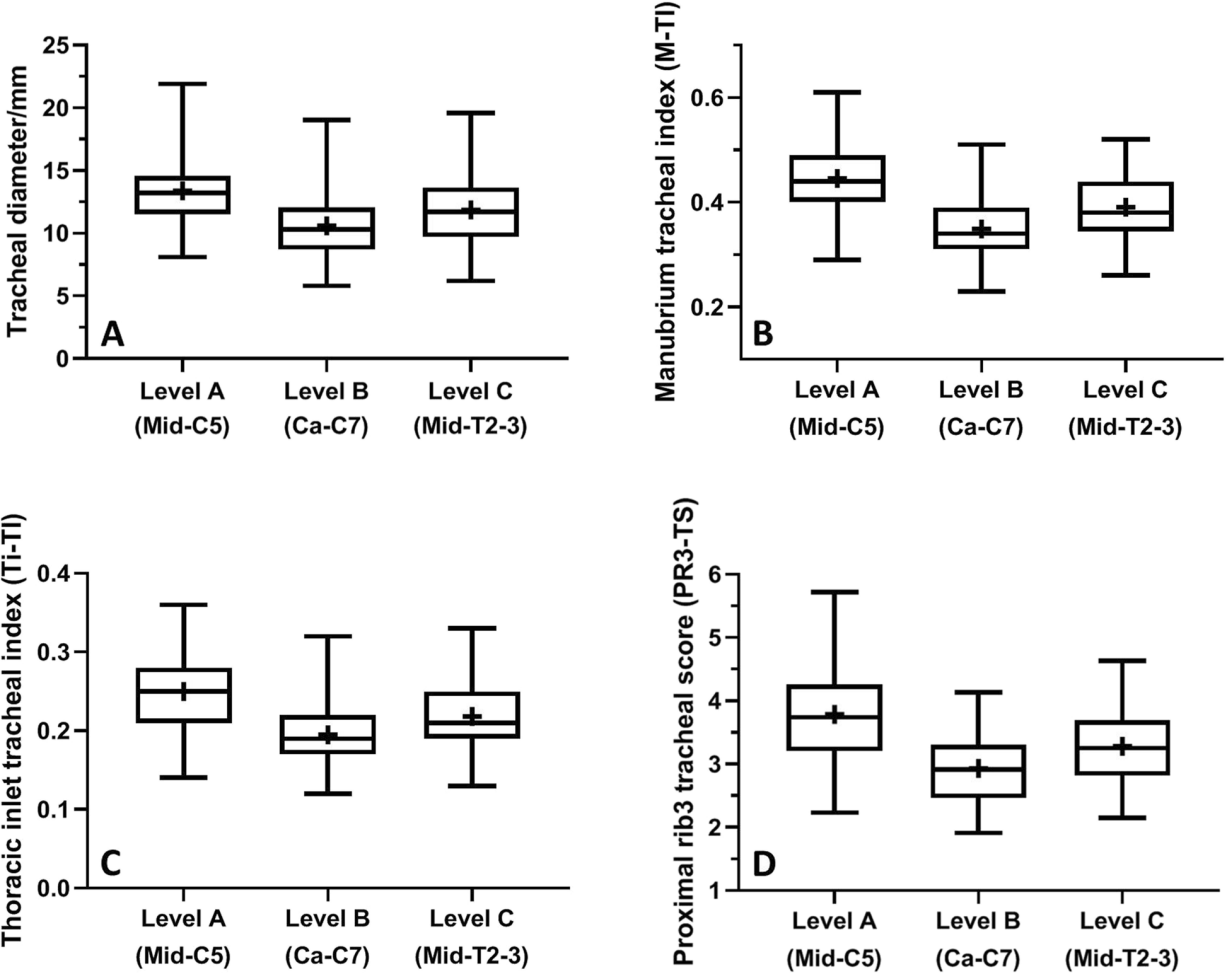
Box-and-whisker plots of the tracheal diameter (**A**), manubrium tracheal index (**B**), thoracic inlet tracheal index (**C**), and proximal rib3 tracheal score (**D**) at levels **A**, **B**, and **C** along the trachea for non-brachycephalic small breed dogs (*n* = 81) with no clinical or radiographic evidence of respiratory or cardiovascular diseases. Boxes and whiskers represent the 25th to 75th percentiles and ranges, respectively; the lines and crosses within boxes represent the medians and means, respectively

Significant differences (*P* < 0.0001) were identified among the mean values of the absolute and standardized tracheal diameters at levels A, B, and C, with the lowest means vertical tracheal diameter (VTD) being noted at the level of the thoracic inlet (Level B). The greatest difference (*P* < 0.0001) was found between the mean values of caudal cervical tracheal diameter (level A) and thoracic inlet tracheal diameter (level B) (Table 1, Fig. 2). The mean VTD calculated at the level of the thoracic inlet (level B, 10.6 mm) was 20.9 and 10.9% lower than those calculated at the levels of caudal cervical (level A, 13.4 mm) and intrathoracic (level C, 11.9 mm) regions, respectively.

**Table 1:** Mean (±SD) values and 95% confidence intervals for radiographic measurements of the absolute, average, and normalized values of vertical tracheal diameter (VTD) at levels A (mid-C5), B (Ca-C7), and C (mid-T2–3) for 81 ‘healthy’ non-brachycephalic, small breed dogs

Variables	Mean ± SD	95% CI	***P***-value < 0.05		
**Absolute vertical tracheal diameter (VTD)/mm**			**ANOVA test**	**Tukey’s test**	**Unpaired t-test**
Level A (Mid-C5), caudal cervical VTD	13.4 ± 2.9	12.7–14.1		A-B, *P* < 0.0001	A-B, *P* < 0.0001
Level B (Ca-C7), thoracic inlet VTD	10.6 ± 2.7	10.0–11.2	*P* < 0.0001	A-C, *P* = 0.004	A-C, *P* = 0.002
Level C (Mid-T2–3), intrathoracic VTD	11.9 ± 3.0	11.2–12.5		B-C, *P* = 0.014	B-C, *P* = 0.005
**Average VTD**	**11.9 ± 2.8**	**11.3–12.6**			
**Normalizing parameter/mm**					
Manubrium length (ML)	30.6 ± 7.4	28.9–32.2			
Thoracic inlet-distance (Ti-D)	54.6 ± 10.6	52.3–57.0			
Proximal R3-width (PR3-W)	3.7 ± 0.8	3.5–3.8			
**Manubrium-tracheal index (M-TI)**			**ANOVA test**	**Tukey’s test**	**Unpaired t-test**
M-TI (Level A)	0.45 ± 0.07	0.43–0.46		A-B, *P* < 0.0001	
M-TI (Level B)	0.35 ± 0.06	0.34–0.36		A-C, *P* < 0.0001	
M-TI (Level C)	0.39 ± 0.06	0.38–0.40		B-C, *P* ≤ 0.0001	
**Average M-TI**	**0.40 ± 0.05**	**0.38–0.41**			
**Thoracic inlet-tracheal index (Ti-TI)**					
Ti-TI (Level A)	0.25 ± 0.04	0.24–0.26		A-B, *P* < 0.0001	A-B, *P* < 0.0001
Ti-TI (Level B)	0.19 ± 0.04	0.19–0.20	*P* < 0.0001	A-C, *P* < 0.0001	A-C, *P* < 0.0001
Ti-TI (Level C)	0.22 ± 0.04	0.21–0.23		B-C, *P =* 0.0005	B-C, *P* ≤ 0.0001
**Average Ti-TI**	**0.22 ± 0.04**	**0.21–0.23**			
**Proximal R3-tracheal score (PR3-TS)**					
PR3-TS (Level A)	3.8 ± 0.7	3.6–4.0		A-B, *P* < 0.0001	
PR3-TS (Level B)	2.9 ± 0.5	2.8–3.1		A-C, *P* < 0.0001	
PR3-TS (Level C)	3.3 ± 0.6	3.2–3.4		B-C, *P =* 0.0009	
**Average PR3-TS**	**3.4 ± 0.6**	**3.2–3.5**

**Fig. 3:**
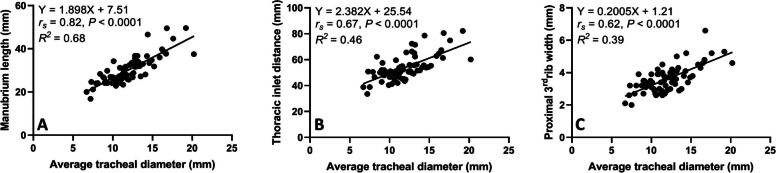
Scatter plots of the average tracheal diameter versus manubrium length (**A**), thoracic inlet distance (**B**), and proximal 3rd rib width (**C**) determined for 81 non-brachycephalic small breed dogs with no clinical or radiographic evidence of respiratory or cardiovascular diseases

**Fig. 4:**
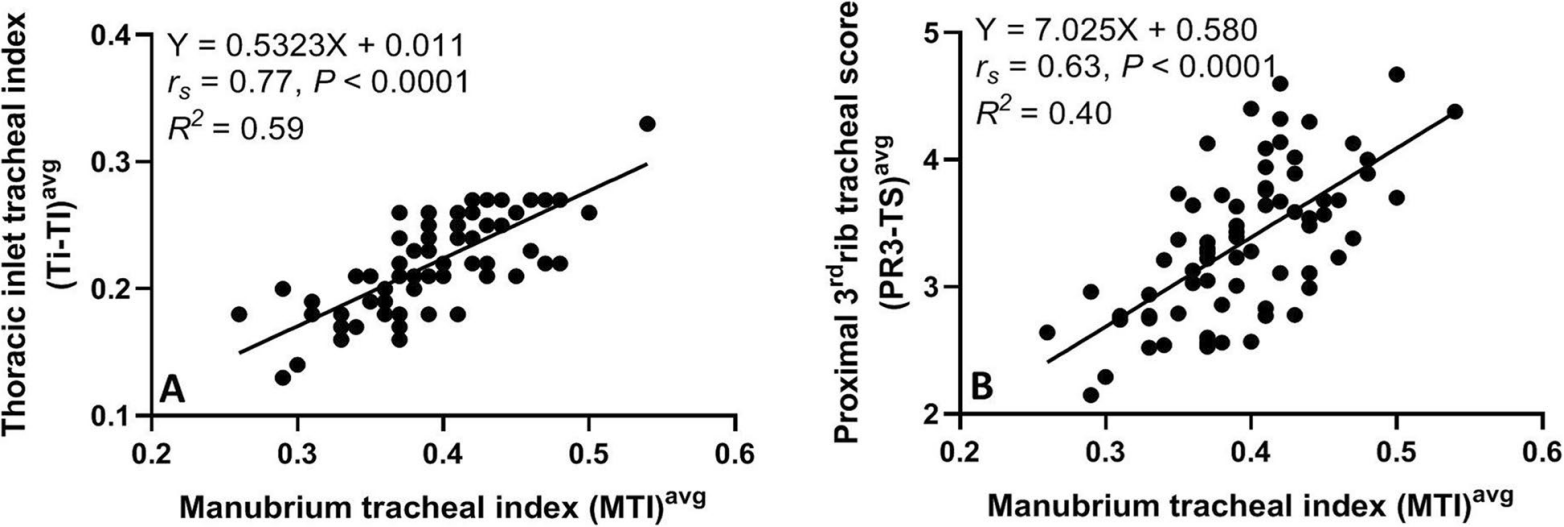
Scatterplots of the average manubrium tracheal index (M-TI) versus average thoracic inlet tracheal index (**A**) and average proximal 3rd rib tracheal score (**B**) determined for 81 non-brachycephalic small breed dogs with no clinical or radiographic evidence of respiratory or cardiovascular diseases

The average tracheal diameter correlated better with the length of the manubrium (*r*_*s*_ = 0.82, *P* < 0.0001) compared to thoracic inlet distance (*r*_*s*_ = 0.67, *P* < 0.0001) and proximal 3rd rib width (*r*_*s*_ = 0.62, *P* < 0.0001) (Fig. 3). A relatively stronger correlation (*r*_*s*_ = 0.77, *P* < 0.0001) was identified between the averaged manubrium tracheal index (M-TI) technique and thoracic inlet tracheal index (Ti-TI) procedure compared to correlation (*r*_*s*_ = 0.63, *P* < 0.0001) calculated between the averaged M-TI and proximal 3rd rib tracheal score (PR3-TS) techniques (Fig. 4). A significant correlation was also identified between the averaged Ti-TI and PR3-TS (*r*_*s*_ = 0.68, *P* < 0.0001).

## Discussion

The main findings of the present study were: 1) vertical diameters of tracheal lumen differed significantly (*P* < 0.0001) according to tracheal region, with the greatest difference being noted between caudal cervical and thoracic inlet tracheal diameters; 2) the narrowest tracheal diameter was identified at the level of the thoracic inlet. Vertical tracheal diameter (VTD) measured at thoracic inlet region was 20.9 and 10.9% narrower than VTDs measured at caudal cervical and intrathoracic regions, respectively; 3) the average VTD correlated better with the manubrium length (*r*_*s*_ = 0.82) compared to thoracic inlet distance (*r*_*s*_ = 0.67) and proximal 3rd rib width (*r*_*s*_ = 0.62); 4) manubrium tracheal index (M-TI) correlated better with thoracic inlet tracheal index (Ti-TI, *r*_*s*_ = 0.77) compared to proximal 3rd rib tracheal score (PR3-TS, *r*_*s*_ = 0.63); 5) means M-TI at levels A, B, and C were 0.45, 0.35, and 0.39, with concomitant 95% CIs being 0.43–0.46, 0.34–0.36, and 0.38–0.40, respectively.

The reported research is innovative and provides a significant contribution to the scientific literature. The study investigated different breeds of clinically and radiographically normal non- brachycephalic dogs. The study demonstrated radiographic evaluation of manubrium-tracheal index (M-TI) at caudal cervical, thoracic inlet, and intrathoracic trachea and determination of the correlation between M-TI and the other conventional procedures. The present study is expected to establish a screening diagnostic protocol for canine tracheal hypoplasia in non-brachycephalic breeds. This could be beneficial to veterinary practitioners through providing key diagnostic and prognostic criteria of tracheal hypoplasia that could be of value in selecting an efficient medical or surgical intervention. Therefore, the authors believe that this article would be of interest to the veterinary clinical practitioners, veterinary surgeons and radiologists as radiography is the most accessible imaging modality worldwide and investigation does not require general anesthesia. Future similar investigation will thus be designed by the same authors to radiographically evaluate the reported standardized VTDs in “normal” versus dyspneic brachycephalic and non-brachycephalic small and large breed dogs.

Several radiographic and CT procedures have been described to determine the tracheal luminal diameter and subsequently diagnose congenital tracheal hypoplasia and acquired tracheal stenosis in dogs [2, 5–10]. In addition to radiography, fluoroscopy and bronchoscopy were established to diagnose and monitor tracheal collapse and hypoplasia in dogs [8, 11]. Although radiographic measurements of canine VTD underestimated tracheal size by an average of ~ 1.0 mm compared to CT [8], radiography remains the most widely used modality among practitioners to evaluate tracheal size in dogs [12, 13]. The rush for radiographic assessment of canine tracheal diameter was reported to be the necessity for surgery and for selecting the proper sizes of the tracheal stent and endotracheal tube [8, 12, 13]. Our investigated thoracic radiographs were assumed to be taken at peak inspiration and without sedation or anesthesia. Nevertheless, unlike dogs with tracheal collapse, those with tracheal hypoplasia don’t reveal a change in the tracheal diameter with the phase of respiration or with positioning for radiography [16]. Radiographic calculation of tracheal index or score was routinely accomplished in dogs at the level of thoracic inlet using thoracic inlet distance (i.e. Ti-TI) or proximal 3rd rib width (i.e. PR3-TS), respectively [2, 6, 7, 14–16]. However, the widths of the proximal 3rd pair of ribs did not always appear equal on the lateral thoracic radiographs, and this could be related to the minimal dog rotation during radiographic positioning [2, 6, 22] which may have biased the results of the PR3-TS. Furthermore, the use of Ti-D was reported to be less susceptible to errors compared to the too small 3rd rib width [6]. Another limitation of the PR3-TS procedure can be the superimposition of the 3rd pair of the ribs that may limit the measurement of their proximal widths, particularly in Bulldogs with thoracic vertebral anomaly and associated crowded ribs. Similarly, the value of thoracic inlet distance (Ti-D) is expected to be influenced by the presence of thoracic vertebral anomaly, as well as by the different locations of the landmarks previously utilized to measure the Ti-D. Therefore, our modified Ti-D was consistently measured from the cranio-ventral aspect of the 1st thoracic vertebra to the highest point of the cranial manubrium, the point of the minimum distance of the thoracic inlet. However, the existence of thoracic vertebral anomaly could also influence the results of the Ti-TI procedure especially in Bulldogs.

Thus, the current study provided the first evidence of radiographic assessment of manubrium tracheal index (M-TI) at three different levels along the canine trachea (caudal cervical, thoracic inlet, and intrathoracic tracheal levels, “A-C”). The caudal cervical tracheal region was selected to be at the level of mid-C5 as it is most likely located at the midway between the caudal aspect of the cricoid cartilage and the thoracic inlet. Besides, this region is commonly included within the collimation of the canine thoracic radiograph. Despite redundant dorsal tracheal membrane was an incidental finding among the clinically and radiographically ‘normal’ dogs, subjects with radiographic evidence of redundant dorsal tracheal membrane were excluded to overcome the possible change in the tracheal lumen during respiration.

In the present study, the mean VTD calculated at the level of the thoracic inlet (level B, 10.6 mm) was 20.9 and 10.9% lower than the means VTD calculated at the levels of caudal cervical (level A, 13.4 mm) and intrathoracic (level C, 11.9 mm) tracheal regions, respectively. These ratios were greater than those previously calculated for large breed dogs; in which, the mean VTD calculated at the level of the thoracic inlet (15.3 mm) was 5.7 and 7.6% smaller than those calculated at the levels of caudal cervical (16.2 mm) and intrathoracic (16.5 mm) tracheal regions, respectively [23]. This indicates the relatively narrower thoracic inlet tracheal diameter in non-brachycephalic small breed dogs compared to large breed dogs. These findings are consistent with a previous study where the diameter and thickness of the tracheal rings were narrowest at the thoracic inlet region [23]. This was explained by the change of the direction of the trachea at the thoracic inlet region which is relatively small and surrounded by bones [23]. Furthermore, the esophagus compresses the trachea and alters its diameter at this level, which may predispose to further thoracic inlet tracheal collapse [23–26]. The authors would even expect much narrower standardized thoracic inlet VTD in brachycephalic versus non-brachycephalic dogs. However, further investigation on a large scale of brachycephalic small and large breed dogs is still warranted to confirm this statement.

The reference values of the thoracic inlet-tracheal index (Ti-TI) previously calculated at the level of the thoracic inlet were 0.11, 0.12 or 0.13 in bulldogs, 0.16 in non-bulldog brachycephalic breeds, and 0.20 or 0.21 in non-brachycephalic small breed dogs (Harvey and Fink 1982 [2, 15, 16, 27]. The previously reported mean Ti-TI value (0.20) calculated at the level of the thoracic inlet in non-brachycephalic dogs is relatively consistent with our reported Ti-TI value (0.19). A Ti-TI value below each of the corresponding numbers reported by our or previous studies would indicate tracheal hypoplasia. The minimal discrepancy in the Ti-TI values between our and previous reports could be related to the minimal variation in outlining the thoracic inlet distance (Ti-D, the minimum distance of the thoracic inlet) measured in the present study, as well as the relatively larger population of healthy non-brachycephalic breeds investigated here. The authors would therefore recommend using a Ti-TI value of 0.19 as a reference value to radiographically diagnose tracheal hypoplasia in non-brachycephalic dogs. The proximal 3rd rib-tracheal score (PR3-TS) previously calculated for intrathoracic trachea (level C) was reported to be greater than 3 [16, 28, 29]. A similar score was determined in the current study, in which the PR3-TS calculated for intrathoracic tracheal diameter (level C) was 3.3. To the authors’ knowledge, assessment of the tracheal index at the caudal cervical region (level A) was not previously established in dogs using different normalizing parameters. Thus, the means M-TI, Ti-TI, and PR3-TS calculated at the caudal cervical trachea (level A) were 0.45, 0.25, and 3.8 in our investigated non-brachycephalic dogs, respectively.

The strong correlation identified between the average tracheal diameter and ML (*r*_*s*_ = 0.82), and the moderate correlation between M-TI and each of Ti-TI (*r*_*s*_ = 0.77) and PR3-TS (*r*_*s*_ = 0.63) would support the use of M-TI as an alternative measure to assess VTD at the three reported tracheal levels in non-brachycephalic small breed dogs. Furthermore, the diagnostic value of both Ti-TI and PR3-TS was reported to be questionable due to the poor agreement identified between the two methods that may have influenced their reliability [7]. Despite the ease of outlining the landmarks of the previously established ML and VTD measurements to calculate M-TI, lack of assessment of inter- and intra-observer variability of the M-TI remains a limitation of the present study. The retrospective nature of the study did not allow for assessment of VTD at the level of the cranial and mid cervical tracheal regions on cervical radiography, which may be a potential limitation of the study. Another limitation is that the investigation of the present study focused solely on healthy non-brachycephalic small breed dogs. Therefore, future studies will be directed to evaluate our reported standardized VTDs in “normal” versus dyspneic brachycephalic and non-brachycephalic small and large breed dogs.

## Conclusions

Vertical tracheal luminal diameters measured along the canine trachea varied according to the location of the tracheal segment. The greatest difference was identified between the caudal cervical and thoracic-inlet tracheal diameters. M-TI could be an appropriate alternative to Ti-TI and PR3-TS for radiographic assessment of canine tracheal diameter at 3 different levels. An average M-TI, Ti-TI, or PR3-TS less than 0.38, 0.21, or 3.2, respectively, may indicate tracheal hypoplasia in non-brachycephalic small breed dogs. A screening protocol, similar to that of canine coxofemoral joint, could be established using the M-TI procedure to assess VTD at 3 different levels along the canine trachea (i.e. VTD scheme).

## Data Availability

The data sets supporting our results are included in the article. Row data are available upon request to the any of the authors (AM: aymostafa@cu.edu.eg; CB: crberry3@ncsu.edu).

## Abbreviations

VTD: Vertical tracheal diameter
ML: Manubrium length
Ti-D: Thoracic inlet distance
PR3-W: Proximal 3rd rib width
M-TI: Manubrium-tracheal index
Ti-TI: Thoracic inlet-tracheal index
PR3-TS: Proximal 3rd rib-tracheal score
